# *Escherichia coli* O104 in Feedlot Cattle Feces: Prevalence, Isolation and Characterization

**DOI:** 10.1371/journal.pone.0152101

**Published:** 2016-03-24

**Authors:** Pragathi B. Shridhar, Lance W. Noll, Xiaorong Shi, Natalia Cernicchiaro, David G. Renter, J. Bai, T. G. Nagaraja

**Affiliations:** 1 Department of Diagnostic Medicine and Pathobiology, Kansas State University, Manhattan, Kansas, United States of America; 2 Veterinary Diagnostic Laboratory, Kansas State University, Manhattan, Kansas, United States of America; U. S. Salinity Lab, UNITED STATES

## Abstract

*Escherichia coli* O104:H4, an hybrid pathotype of Shiga toxigenic and enteroaggregative *E*. *coli*, involved in a major foodborne outbreak in Germany in 2011, has not been detected in cattle feces. Serogroup O104 with H type other than H4 has been reported to cause human illnesses, but their prevalence and characteristics in cattle have not been reported. Our objectives were to determine the prevalence of *E*. *coli* O104 in feces of feedlot cattle, by culture and PCR detection methods, and characterize the isolated strains. Rectal fecal samples from a total of 757 cattle originating from 29 feedlots were collected at a Midwest commercial slaughter plant. Fecal samples, enriched in *E*. *coli* broth, were subjected to culture and PCR methods of detection. The culture method involved immunomagnetic separation with O104-specific beads and plating on a selective chromogenic medium, followed by serogroup confirmation of pooled colonies by PCR. If pooled colonies were positive for the *wzx*_O104_ gene, then colonies were tested individually to identify *wzx*_O104_-positive serogroup and associated genes of the hybrid strains. Extracted DNA from feces were also tested by a multiplex PCR to detect *wzx*_O104_-positive serogroup and associated major genes of the O104 hybrid pathotype. Because *wzx*_O104_ has been shown to be present in *E*. *coli* O8/O9/O9a, *wzx*_O104_-positive isolates and extracted DNA from fecal samples were also tested by a PCR targeting *wbdD*_O8/O9/O9a_, a gene specific for *E*. *coli* O8/O9/O9a serogroups. Model-adjusted prevalence estimates of *E*. *coli* O104 (positive for *wzx*_O104_ and negative for *wbdD*_O8/O9/O9a_) at the feedlot level were 5.7% and 21.2%, and at the sample level were 0.5% and 25.9% by culture and PCR, respectively. The McNemar’s test indicated that there was a significant difference (*P* < 0.01) between the proportions of samples that tested positive for *wzx*_O104_ and samples that were positive for *wzx*_O104_, but negative for *wbdD*_O8/O9/O9a_ by PCR and culture methods. A total of 143 isolates, positive for the *wzx*_O104_, were obtained in pure culture from 146 positive fecal samples. Ninety-two of the 143 isolates (64.3%) also tested positive for the *wbdD*_O8/O9/O9a_, indicating that only 51 (35.7%) isolates truly belonged to the O104 serogroup (positive for *wzx*_O104_ and negative for *wbdD*_O8/O9/O9a_). All 51 isolates tested negative for *eae*, and 16 tested positive for *stx*1 gene of the subtype 1c. Thirteen of the 16 *stx*1-positive O104 isolates were from one feedlot. The predominant serotype was O104:H7. Pulsed-field gel electrophoresis analysis indicated that *stx*1-positive O104:H7 isolates had 62.4% homology to the German outbreak strain and 67.9% to 77.5% homology to human diarrheagenic O104:H7 strains. The 13 isolates obtained from the same feedlot were of the same PFGE subtype with 100% Dice similarity. Although cattle do not harbor the O104:H4 pathotype, they do harbor and shed Shiga toxigenic O104 in the feces and the predominant serotype was O104:H7.

## Introduction

In the summer of 2011, Germany and other European countries experienced a large outbreak of foodborne illness affecting nearly 4,000 people, with about 900 developing hemolytic uremic syndrome, leading to 54 deaths [1]. The causative agent was identified as *Escherichia coli* O104:H4, a hybrid serotype possessing characteristics of two pathotypes of *E*. *coli*, Shiga toxin-producing *E*. *coli* (STEC) and enteroaggregative *E*. *coli* (EAEC). The outbreak strain carried Shiga toxin 2 gene (*stx*2) and genes characteristic of EAEC, such as *aatA* (pAA virulence plasmid marker gene), *aggA* (pilin subunit of aggregative adherence fimbriae I), *aggR* (aggregative adherence fimbriae I transcriptional regulator), but was negative for other enterohemorrhagic *E*. *coli* (EHEC) genes, such as *stx*1 (Shiga toxin 1), *eae* (intimin) and *ehxA* (enterohemolysin) [2]. The serogroup O104 has serotypes other than O104:H4 and at least two of them have been implicated in human illnesses. A *stx*2-carrying O104:H21 serotype that was also negative for *eae* (similar to the O104:H4 German strain) was implicated in an outbreak of hemorrhagic colitis associated with consumption of raw milk in Helena, Montana in 1994 [3]. Sporadic cases of diarrhea caused by O104:H7, carrying *stx*1 or *stx*2, also negative for *eae*, have been reported [4, 5]. Non-Shiga toxigenic strains of *E*. *coli* O104 that were either EAEC or enteropathogenic (EPEC) pathotype have been reported in human patients with diarrhea in South Africa [6].

Because cattle are a primary reservoir of STEC, studies have been conducted to determine whether cattle harbor the O104:H4 serotype. Wieiler et al. [7] tested 100 cattle fecal samples from 34 different farms in the outbreak region of Germany and found that none of the fecal samples were positive for *E*. *coli* strains carrying genes characteristic of O104:H4. Auvray et al. [8] analyzed 1,468 cattle fecal samples collected from several slaughter facilities in France by real-time and conventional PCR assays that targeted *stx*2, *wzx*_O104_, *fliC*_H4_ (H4 flagellar gene) and *aggR* and reported that none of the fecal samples were positive for all four genes. We conducted a study to detect *E*. *coli* O104:H4 in feedlot cattle fecal samples (n = 248) using a multiplex PCR that targeted O104 (*wzx*_O104_), H4 (*fliC*_*H4*_), aggregative adherence fimbriae 1 (*aggA*), Shiga toxins 1 and 2 (*stx*1 and *stx*2), intimin (*eae*), tellurite resistance (*terD*), and enterohemolysin (*ehxA*), characteristic of the outbreak serotype and reported that cattle feces were positive for the O104 serogroup, but negative for the hybrid pathotype [9]. In that study, fecal samples positive for *wzx*_O104_ were plated onto several selective and differential media, and only a small number of PCR-positive samples yielded pure cultures of serogroup O104 and none of the isolates carried Shiga toxin genes. The likely reason for the poor recovery of O104 from PCR-positive fecal samples was that the culture method did not have an immunomagnetic separation (IMS) step because O104-specific IMS beads were not available at the time the study was conducted. Therefore, our objectives were to determine the prevalence of *E*. *coli* O104 in feedlot cattle feces utilizing a culture method involving an IMS step and a PCR-based method of detection, and to characterize the isolated strains. The culture method utilized in the study involved an enrichment step, followed by IMS with O104-specific IMS beads, plating on a chromogenic selective medium and confirming the O104 serogroup and major virulence genes by a multiplex PCR. The *wzx*_O104_ gene that was targeted in the PCR assay to detect O104 has also been reported in O8, O9 and O9a serogroups of *E*. *coli* [10]. Therefore, pre- and post-enriched fecal suspensions and putative *E*. *coli* O104 isolates were also subjected to a PCR assay with primers that targeted *wbdD* (which codes for methyl and kinase transferase), specific for *E*. *coli* O8, O9 and O9a serogroups [11].

## Materials and Methods

### Sample collection

The study was approved by the Kansas State University Institutional Animal Care and Use Committee (IACUC # 3172). Rectal content samples were collected from feedlot cattle immediately after slaughter at a Midwest slaughter plant during two visits, one week apart, in July 2013. The permission to collect samples was given under an agreement that the name and location of the abattoir will not be disclosed. Rectums were incised and contents were scooped with a plastic spoon. The spoon with the contents (approximately 10 to 20 g) were placed in a Whirl-Pak bag (Nasco, Ft. Atkinson, WI), transported on ice in a cooler to the Preharvest Food Safety Laboratory at Kansas State University and processed within 24 h. Rectal content samples were collected from a total of 757 cattle (both heifers and steers) originating from 29 feedlots located in six Midwestern States (IA, IL, MN, MO, NE and SD). Sixteen to 38 samples were collected per lot of cattle from a total of 35 lots, with each lot consisting of 36 to 227 animals. Cattle from one feedlot (No. 2) were sampled in both weeks.

### Culture method of detection

Approximately two grams of fecal samples were suspended in 18 ml of *E*. *coli* broth (EC; Difco^™^, Becton, Dickinson Co., Sparks, MD) and incubated at 40°C for 6 h [9]. Post-enrichment fecal samples were subjected to a culture-based procedure, which involved immunomagnetic separation with O104 serogroup-specific beads (Abraxis^®^, Warminster, PA), and plating onto a selective chromogenic Possé medium [12] modified to include novobiocin at 5 mg/l and potassium tellurite at 0.5 mg/l (MP) [13]. After 20–24 h of incubation at 37°C, six chromogenic colonies (mauve, pink, or purple) were picked and streaked onto blood agar and incubated at 37°C for 24 h. The six colonies were pooled, boiled and the lysate was subjected to an eight-plex PCR targeting O-antigen genes of O104 (*wzx*_O104_) and the seven major serogroups of STEC (O26, O45, O103, O111, O121, O145 and O157). If pooled colonies were positive for *wzx*_O104_, then the six colonies were tested individually by a nine-plex PCR to identify pure culture of putative O104 serogroup (*wzx*_O104_) and associated major genes of the STEC and EAEC pathotypes: *stx*1 (Shiga toxin 1), *stx*2 (Shiga toxin 2), *eae* (intimin), *ehxA* (enterohemolysin), *terD* (tellurite resistance), *aggA* (pilin subunit of aggregative adherence fimbriae 1), *bfpA* (bundle-forming pilus) and *flic*_H4_ (H4-specific flagella). The primer pairs used for the eight-plex and nine-plex PCR assays are listed in Table 1. PCR amplification protocol for both assays included an initial denaturation at 94°C for 5 min followed by 25 cycles (pure culture) or 35 cycles (fecal suspension in broth) of 94°C for 30 s, 65°C for 30 s, 68°C for 75 s, and the final extension was 68°C for 7 min [9]. Isolates confirmed as positive for the *wzx*_O104_ gene were stored on cryogenic beads (CryoCare^™^, Key Scientific Products, Round Rock, TX).

**Table 1:** Target genes, primer sequences used and size of amplicons in PCR assays.

Target gene	Primer sequences	Amplicon size (bp)	Reference
***wzx***_**O26**_	F:AGGGTGCGAATGCCATATT	417	[33]
	R:GACATAATGACATACCACGAGCA		
***wzx***_**O45**_	F: GGGCTGTCCAGACAGTTCAT	890	[33]
	R: TGTACTGCACCAATGCACCT		
***wzx***_**O103**_	F: GCAGAAAATCAAGGTGATTACG	740	[33]
	R: GGTTAAAGCCATGCTCAACG		
***wzx***_**O104**_	F: GGTTTTATTGTCGCGCAAAG	337	[9]
	R: TATGCTCTTTTTCCCCATCG		
***wzx***_**O111**_	F: ACAAGAGTGCTCTGGGCTTC	230	[13]
	R: AAACTAAGTGAGACGCCACCA		
***wbq*E + *wbq*F**_**O121**_	F: TCAGCAGAGTGGAACTAATTTTGT	587	[33]
	R: TGAGCACTAGATGAAAAGTATGGCT		
***wzx***_**O145**_	F: TCAAGTGTTGGATTAAGAGGGATT	523	[33]
	R: CACTCGCGGACACAGTACC		
***rfb*E**_**O157**_	F: CAGGTGAAGGTGGAATGGTTGTC	296	[34]
	R: TTAGAATTGAGACCATCCAATAAG		
***wbdD***_**O8/O9/O9a**_	F: GGCATCGGTCGGTATTCC	1000	[11]
	R: TGCGCTAATCGCGTCTAC		
***stx*1**	F: TGTCGCATAGTGGAACCTCA	655	[34]
	R: TGCGCACTGAGAAGAAGAGA		
***stx*2**	F: CCATGACAACGGACAGCAGTT	477	[34]
	R: TGTCGCCAGTTATCTGACATTC		
***eae***	F: CATTATGGAACGGCAGAGGT	375	[34]
	R: ACGGATATCGAAGCCATTTG		
***ehxA***	F: GCGAGCTAAGCAGCTTGAAT	199	[34]
	R: CTGGAGGCTGCACTAACTCC		
***terD***	F: AGTAAAGCAGCTCCGTCAAT	434	[2]
	R: CCGAACAGCATGGCAGTCT		
***aggA***	F: CGTTACAAATGATTGTCCTGTTACTAT	151	[9]
	R: ACCTGTTCCCCATAACCAGAC		
***bfpA***	F: CAGAAGTAATGAGCGCAACG	285	This study
	R: CGTAGCCTTTCGCTGAAGTA		
***flic***_**H4**_	F: ACGGCTGCTGATGGTACAG	244	[9]
	R: CGGCATCCAGTGCTTTTAAC		
***flic***_**H2**_	F: GCAACGGCTGAAACAACCTA	585	This study
	R: TGCAGTTACAACTTCGGTTTTG		
***flic***_**H4**_	F: ACGGCTGCTGATGGTACAG	244	[9]
	R: CGGCATCCAGTGCTTTTAAC		
***flic***_**H7**_	F: AGCTGCAACGGTAAGTGATTT	949	[34]
	R: GGCAGCAAGCGGGTTGGTC		
***flic***_**H11**_	F: TCTGACACAAACATAGCTGGTACA	228	This study
	R: TGTCTCACTCGTAATCAAAGAAGC		
***flic***_**H21**_	F: TCGATGGCGCGCAGAAAGCA	419	[35]
	R: GGCTGTCGTAGGGGCAACGG		

### PCR method of detection

One ml of pre- and post-enriched fecal suspensions were removed, boiled for 10 min and centrifuged at 9,300 x g for 5 min. The DNA in the supernatant was purified using a GeneClean^®^ Turbo Kit (MP Biomedicals LLC., Solon, OH). DNA extracted from pre- and post-enrichment samples were tested by a nine-plex PCR to detect O antigen of O104 serogroup (*wzx*_O104_) and associated major genes of the STEC and EAEC (*stx*1, *stx*2, *eae*, *ehxA*, *terD*, *aggA*, *bfpA* and *flic*_H4_) and a single-plex PCR assay to detect *wbdD*_O8/O9/O9a_, a gene specific for serogroups O8, O9 and O9a [11].

### Characterization of the *E*. *coli* O104 isolates

All putative O104 isolates (positive for *wzx*_O104_) were tested by a PCR assay targeting O8/O9/O9a (*wbdD*_O8/O9/O9a_), and isolates positive for *wzx*_O104_ and negative for *wbdD*_O8/O9/O9a_ were considered as truly O104, and were further characterized. The flagellar types of the O104 isolates were identified by a multiplex PCR targeting five flagellar types (H2, H4, H7, H11 and H21) (Table 1) using the PCR running conditions as described before for the nine- or eight-plex PCR. The subtype of Shiga toxin genes was identified by nucleotide sequencing. Shiga toxin genes of the *wzx*_O104_-positive isolates were amplified (F-GCTCAAGGAGTATTGTGTAATATG and R- TCGCTGAATCCCCYTC) by a touchdown PCR method where the annealing temperature of each cycle was lowered gradually to avoid amplification of non-specific sequences [14]. PCR amplification protocol included an initial denaturation at 94°C for 5 min, 10 cycles of touchdown PCR (denature: 94°C for 30 s, annealing: 56–51°C (Δ-0.5°C) for 30 s; extension: 72°C for 1 min 45 s) followed by 30 cycles of regular PCR (denaturation: 94°C for 30 s, annealing: 51°C for 30 s; extension: 72°C for 1 min 45 s). Amplicons (1,233 bp) were purified using QIAquick^®^ PCR Purification Kit. The purity and concentration of purified PCR products were determined using a spectrophotometer (NanoDrop, Thermo Scientific, Wilmington, DE). The products were then shipped to Genewiz Inc., (South Plainfield, NJ) for sequencing. The sequence data were aligned using the CLC Main Workbench 6.8.4 software for further analysis. Shiga toxin subtypes were determined according to the procedure described by Scheutz et al., [15]. Briefly, nucleotide sequences were translated to amino acid sequences after removing intergenic regions. The subtype of Shiga toxins was determined based on the amino acid motifs that define each Shiga toxin subtype [15]. Additionally, the subtyping of Shiga toxin genes was confirmed by PCR-RFLP (restriction fragment length polymorphism) [16]. Briefly, the B subunit of *stx*1 genes was amplified yielding a 283 bp amplicon. PCR amplification protocol included an initial denaturation at 94.0°C for 5 min, followed by 35 cycles of 94.0°C for 30 s, 51.3°C for 60 s, 72.0°C for 40 s. PCR products were purified using QIAquick^®^ PCR purification Kit, digested separately with *BstE*II, *Hae*II and *Pflm*I enzymes. Restriction fragments were visualized by microcapillary electrophoresis using Qiaxcel (Qiagen, Valencia, CA). Additionally, subtyping of *stx*1was conducted by *in silico* RFLP. A 283 bp fragment starting with CAGTTGAGGGGGGTAAAATG (forward primer used for amplification of *stx*1 in PCR-RFLP) and ending with GATTCAGCGAAGTTATTTTCCG (reverse complement of reverse primer used for amplification of *stx*1 in PCR-RFLP) was digested by *BstE*II, *Hae*II and *Pflm*I using CLC Main Workbench 6.8.4 software. Restriction patterns of O104 STEC isolates were compared to reference sequences of three *stx*1 subtypes from the NCBI database (*stx*1a: Accession no. M16625.1; *stx*1c: Accession no. DQ449666.1; *stx*1d: Accession no. AY170851.1).

### Pulsed-field gel electrophoresis (PFGE)

The clonal relationships between O104 STEC strains isolated from cattle feces were assessed by PFGE typing. The human clinical strains, German (O104:H4; BAA-2326) and Montana outbreak (O104:H21; BAA-178) and O104:H7 strains (06–3637, 07–3598, 08–4061, 2011C-3665, 2012C-3400; provided by Nancy A. Strockbine, Centers for Disease Control and Prevention, Atlanta, GA) were also included for comparison. The PFGE was performed according to the Centers for Disease Control and Prevention’s PulseNet protocol. Briefly, agarose embedded DNA of the O104 isolates were digested with *Xba*I followed by separation of restriction fragments by electrophoresis. *Salmonella enterica* serotype Braenderup (strain H9812) DNA digested with *Xba*I was used as DNA marker. Gel images were captured with a Gel Doc 2000 system (Bio-Rad). PFGE patterns were analyzed using the Bionumerics software (Applied Maths, Inc., Austin, TX). Band-based Dice similarity coefficients and unweighted pair-group method for clustering with a position tolerance setting of 1.5% were used for optimization and band comparison. Isolates with 100% homology were grouped as subtypes and those isolates which were > 96% but less than 100% homologous were grouped as types based on the Dice coefficients.

### Statistical analysis

Generalized linear mixed models (GLMM) were fitted using a binary or binomial (events/trials) distribution, logit link, Laplace estimation, and Newton-Raphson and Ridging optimization, to estimate fecal prevalence of *wzx*_O104_ and *wzx*_O104/_*wbdD*_O8/O9/O9a_ in Proc Glimmix (SAS 9.3, SAS Institute Inc., Cary, NC). Sample-, lot- and feedlot-level prevalence were calculated as the proportion of samples, samples within lots or samples within feedlots testing positive for *wzx*_O104_ (serogroup O104 and or O8/O9/O9a) and positive for *wzx*_O104_ but negative for *wbdD*_O8/O9/O9a_ (serogroup O104 only) by PCR or culture methods, and divided by the total number of samples tested per lot or per feedlot, respectively. Random effects were used to account for the hierarchical structure of the data (samples within lots, lots within feedlots and feedlots within states). Prevalence estimates (and 95% confidence intervals) were obtained from model intercepts using the formula p = e^β0^ / (1 + e^β0^), where β^0^ is the coefficient of the model intercept. The Cohen’s Kappa statistic was used to compute the agreement beyond that due to chance between PCR- and culture-based methods for detection of serogroup O104 and or O8/O9/O9a (positive for *wzx*_O104_) and serogroup O104 only (*wzx*_O104_ positive but negative for *wbdD*_O8/O9/O9a_) in fecal samples. Interpretation of the Kappa statistic was based on the scale proposed by Landis and Koch [17]. The McNemar’s Chi-square test was used to compare the proportion of positive samples detected by both methods [18]. When the *P*-value of the McNemar’s test is not significant (*P* > 0.01), there is little evidence to conclude that the proportion of positives are different, whereas when the *P*-value is significant (*P* < 0.01), there is a significant disagreement between tests, indicating little value in assessing agreement. In the latter case, Cohen’s Kappa statistics are provided for reference only.

## Results

### Culture method of detection

Of the total 757 fecal samples tested, 146 samples (19.3%) tested positive for serogroup O104 (and/or O8/O9/O9a), based on PCR testing (for the *wzx*_O104_ gene) of the six-pooled colonies picked from the O104 IMS beads-inoculated MP plates. Fecal samples from 21 of 29 feedlots (72.4%) and 27 of 35 lots (77.1%) tested positive for the serogroup O104 (and/or O8/O9/O9a) (Table 2). At the sample level, the crude prevalence of serogroup O104 (and or O8/O9/O9a) within feedlots ranged from 0 to 93.8%. Because six chromogenic colonies were picked from O104 beads-plated medium, pooled, and tested by a multiplex PCR targeting a total of eight serogroups (O26, O45, O103, O104, O111, O121, O145, and O157), other *E*. *coli* serogroups were also detected occasionally. Pooled colonies from fecal samples (n = 757) also tested positive for O26 (3.7%), O45 (0.9%), O103 (5.8%), O145 (0.5%) and O157 (4.9%). Serogroups O111 and O121 were not detected in any of these samples. The testing of individual colonies was designed to identify *wzx*_O104_-positive serogroup and associated virulence genes only; therefore, individual isolates of the other seven serogroups were not identified. A total of 143 isolates, positive for the *wzx*_O104_, were obtained in pure culture from 146 positive (based on pooled colonies) fecal samples. Ninety-two of the 143 isolates (64.3%) also tested positive for the *wbdD*_O8/O9/O9a_ (Table 2), indicating that only 51 (35.7%) isolates truly belonged to the O104 serogroup (positive for *wzx*_O104_ and negative for *wbdD*_O8/O9/O9a_). After excluding the samples that yielded isolates positive for O8/O9/O9a, only 14 of the 29 (48.3%) feedlots, 20 of the 35 lots (57.1%), and 49 of the 757 (6.5%) samples were considered truly positive for the serogroup O104.

**Table 2:** Number of fecal samples from feedlot cattle positive for *wzx*_O104_ possessing *E*. *coli* (serogroups O104 and/or O8/O9/O9a) based on the culture method and *wzx*_O104_-positive isolates that tested positive for *wbdD*_O8/O9/O9a_ (serogroups O8/O9/O9a) or negative for *wbdD*_O8/O9/O9a_ (serogroup O104).

					No. of *wzx*_O104_-positive isolates	
Week	Feedlot no.	Lot No.	No. of samples collected	No. of samples positive for *wzx*_O104_ (%)^a^	Positive for *wbdD*_O8/O9/O9a_ (%)^b^	Negative for *wbdD*_O8/O9/O9a_ (%)^b^
**1**	1	1	38	18 (47.4)	20 (52.6)	0
	2	2	38	5 (13.2)	4 (10.5)	2 (5.3)
	2	3	19	3 (15.8)	0	1 (5.3)
	3	4	38	0	0	0
	4	5	19	6 (31.6)	4 (21.1)	2 (10.5)
	4	6	19	9 (47.4)	4 (21.1)	5 (26.3)
	5	7	38	1 (2.6)	0	1 (2.6)
	6	8	19	3 (15.8)	0	3 (15.8)
	7	9	19	11 (57.9)	10 (52.6)	1 (5.3)
	8	10	19	0	0	0
	9	11	19	0	0	0
	10	12	19	0	0	0
	11	13	19	4 (21.1)	0	3 (15.8)
	12	14	19	1 (5.3)	0	0
	13	15	19	8 (42.1)	2 (10.5)	5 (26.3)
	14	16	16	15 (93.8)	16 (100.0)	2 (12.5)
**2**	2	17	20	2 (10.0)	0	2 (10.0)
	2	18	20	5 (25.0)	3 (15.0)	1 (5.0)
	15	19	20	1 (5.0)	0	1 (5.0)
	16	20	20	2 (10.0)	2 (10.0)	0
	17	21	20	1 (5.0)	0	1 (5.0)
	18	22	20	2 (10.0)	0	0
	19	23	20	0	0	0
	20	24	20	2 (10.0)	2 (10.0)	0
	21	25	20	10 (50.0)	9 (45.0)	0
	22	26	20	5 (25.0)	2 (10.0)	3 (15.0)
	22	27	20	5 (25.0)	2 (10.0)	3 (15.0)
	23	28	20	11 (55.0)	11(55.0)	0
	24	29	20	1 (5.0)	0	1 (5.0)
	25	30	20	0	0	0
	26	31	20	9 (45.0)	0	9 (45.0)
	26	32	20	5 (25.0)	1 (5.0)	4 (20.0)
	27	33	20	1 (5.0)	0	1 (5.0)
	28	34	20	0	0	0
	29	35	20	0	0	0
Total	29	35	757	146 (19.3)	92 (12.2)	51 (6.7)

^a^Samples positive by PCR of pooled colonies

^b^The percentages in parentheses are number of *wzx*_O104_ isolates that were positive or negative for *wbdD*_O8/O9/O9a_ from the total number of samples in each lot.

### PCR method of detection

Of the nine genes included in the multiplex PCR assay, two genes, *aggA* and *bfpA*, which code for aggregative adherence fimbriae 1 and bundle forming pili, respectively, were not detected in any of the fecal samples, either before or after enrichment in EC broth (Table 3). The overall prevalence of *wzx*_O104_ gene in samples before and after enrichment was 5% (38/757) and 46.1% (349/757), respectively. Based on the single-plex assay of samples targeting *wbdD*_O8/O9/O9a_, 13 (1.7%) and 238 (31.4%) of the 757 samples tested were positive—before and after enrichment, respectively. Thirty-four (4.5%) and 194 (25.6%) fecal samples were positive for *wzx*_O104_ and negative for *wbdD*_O8/O9/O9a_, suggesting that those fecal samples truly contained *E*. *coli* O104 (Table 3). A higher proportion of fecal samples tested positive for the *stx*2 than *stx*1 gene (66.8 vs. 27.5%) in enriched samples. Among the genes tested, *ehxA* that codes for enterohemolysin was the most prevalent in both pre- and post-enriched samples, followed by *flic*_H4_, *terD*, *eae*, *stx*2 and *stx*1 genes (Table 3).

**Table 3:** Number of fecal samples from feedlot cattle positive for *wzx*_O104_ and or *wbdD*_O8/O9/O9a_ and associated major genes of the O104 hybrid pathotype (O104:H4) in cattle feces based on the PCR method.

		No. of samples (n = 757) positive	
Genes	Protein or Function	Before enrichment, n (%)	After enrichment^a^, n (%)
***wzx***_**O104**_	O104 antigen flippase	38 (5.0)	349 (46.1)
***wbdD***_**O8/O9/O9a**_	Kinase and methyl transferase	13 (1.7)	238 (31.4)
**Only**^b^ ***wzx***_**O104**_		34 (4.5)	194 (25.6)
***stx*1**	Shiga toxin 1	29 (3.8)	208 (27.5)
***stx*2**	Shiga toxin 2	156 (20.6)	506 (66.8)
***eae***	Intimin	112 (14.8)	549 (72.5)
***ehxA***	Enterohemolysin	370 (48.9)	710 (93.8)
***terD***	Tellurite resistance	339 (44.8)	624 (82.4)
***aggA***	Aggregative adherence fimbriae 1	0 (0)	0 (0)
***bfpA***	Bundle forming pili	0 (0)	0 (0)
***flic***_**H4**_	H4 flagellar antigen	200 (26.4)	659 (87.1)

^a^ Feces were enriched in *Escherichia coli* broth at 40 C for 6 h.

^b^ Positive for *wzx*_O104_ and negative for *wbdD*_O8/O9/O9a_.

Based on the PCR assay of post-enriched fecal suspensions, 26 of 29 feedlots (89.7%) and 32 of 35 lots (91.4%) contained one or more fecal samples that tested positive for *wzx*_O104_ (serogroup O104 and or O8/O9/O9a). At the sample level, the crude prevalence of *wzx*_*O104*_ within feedlots ranged from 0 to 95%. Of the three feedlots that were negative for *wzx*_O104_, two had samples that tested positive for *wbdD*_O8/O9/O9a_. Twenty-three of the 29 feedlots (79.3%) and 29 of the 35 lots (82.9%) were positive for *wzx*_O104_ and negative for *wbdD*_O8/O9/O9a_, indicating that cattle feces from these feedlots can be considered truly positive for serogroup O104. Five of the 29 feedlots were negative for *wbdD*_O8/O9/O9a_ and of those four were positive for *wzx*_O104_ (Table 4).

**Table 4:** Number of fecal samples from feedlot cattle positive for *E*. *coli* O104 and O8/O9/O9a based on PCR assays of *wzx*_O104_ and *wbdD*_O8/O9/O9a_.

				Positive for:		
Week	Feedlot no.	Lot No.	No. of samples collected	*wzx*_O104_	*wbdD*_O8/O9/O9a_	*wzx*_O104_ and negative for *wbdD*_O8/O9/O9a_
**1**	1	1	38	31 (81.6)	12 (31.6)	21 (55.3)
	2	2	38	10 (26.3)	9 (23.7)	6 (15.8)
	2	3	19	9 (47.4)	7 (36.8)	5 (26.3)
	3	4	38	3 (7.9)	5 (13.2)	2 (5.3)
	4	5	19	14 (73.7)	16 (84.2)	0
	4	6	19	18 (94.7)	7 (36.8)	11 (57.9)
	5	7	38	26 (68.4)	19 (50.0)	12 (31.6)
	6	8	19	7 (36.8)	2 (10.5)	5 (26.3)
	7	9	19	12 (63.2)	5 (26.3)	8 (42.1)
	8	10	19	0	1 (5.3)	0
	9	11	19	0	0	0
	10	12	19	2 (10.5)	0	2 (10.5)
	11	13	19	8 (42.1)	0	8 (42.1)
	12	14	19	5 (26.3)	1 (5.3)	4 (21.1)
	13	15	19	10 (52.6)	1 (5.3)	10 (52.6)
	14	16	16	10 (62.5)	3 (18.8)	8 (50.0)
**2**	2	17	20	5 (25.0)	3 (15.0)	4 (20.0)
	2	18	20	11 (55.0)	3 (15.0)	10 (50.0)
	15	19	20	5 (25.0)	0	5 (25.0)
	16	20	20	14 (70.0)	5 (25.0)	11 (55.0)
	17	21	20	4 (20.0)	1 (5.0)	4 (20.0)
	18	22	20	19 (95.0)	18 (90.0)	1 (5.0)
	19	23	20	13 (65.0)	20 (100.0)	0
	20	24	20	6 (30.0)	5 (25.0)	4 (20.0)
	21	25	20	16 (80.0)	7 (35.0)	9 (45.0)
	22	26	20	18 (90.0)	9 (45.0)	9 (45.0)
	22	27	20	15 (75.0)	12 (60.0)	5 (25.0)
	23	28	20	17 (85.0)	14 (70.0)	3 (15.0)
	24	29	20	1 (5.0)	5 (25.0)	0
	25	30	20	2 (10.0)	15 (75.0)	1 (5.0)
	26	31	20	9 (45.0)	9 (45.0)	4 (20.0)
	26	32	20	9 (45.0)	10 (50.0)	5 (25.0)
	27	33	20	4 (20.0)	13 (65.0)	1 (5.0)
	28	34	20	0	1 (5.0)	0
	29	35	20	16 (80.0)	0	16 (80.0)
Total	29	35	757	349 (46.1)	238 (31.4)	194 (25.6)

Model-adjusted prevalence estimates of *wzx*_O104_ -positive fecal samples (serogroups O104 and/or O8/O9/O9a) and *wzx*_O104_ -positive fecal samples that were negative for *wbdD*_O8/O9/O9a_ (serogroup O104 only) at the sample-, lot- and feedlot-levels detected by culture and PCR methods are presented in Table 5. The McNemar’s test indicated that there was a significant difference (*P* < 0.01) between the proportions of samples that tested positive for *wzx*_O104_ and samples that were positive for *wzx*_O104_, but negative for *wbdD*_O8/O9/O9a_ by PCR and culture methods, hence the Kappa statistics (κ = 0.27; κ 95% CI = 0.21–0.32 for samples that tested positive for *wzx*_O104_ and κ = 0.10; κ 95% CI = 0.04–0.17 for samples that were positive for *wzx*_O104_, but negative for *wbdD*_O8/O9/O9a_) are provided for reference only.

**Table 5:** Model-adjusted prevalence estimates of fecal samples from feedlot cattle positive for *wzx*_O104_/*wbdD*_O8/O9/O9a_ and *wzx*_O104_ at the feedlot-, lot- and sample- levels.

Target genes, serogroups, and detection method	Level of prevalence estimation	Mean Prevalence, % (95% confidence interval)
**Positive for *wzx***_**O104**_ **(Positive for O104 and/or O8/O9/O9a)** ^a^^,^ ^b^		
**Culture method**	Feedlot	17.6 (6.3–40.3)
	Lot	13.5 (4.8–32.9)
	Sample	11.8 (6.3–21.0)
**PCR method**	Feedlot	49.5 (29.3–69.9)
	Lot	42.5 (22.6–65.1)
	Sample	41.7 (27.1–57.8)
**Positive for *wzx***_**O104**_ **and negative for *wbdD***_**O8/O9/O9a**_ **(Positive for O104 only)** ^a^^,^ ^c^		
**Culture method**	Feedlot	5.7 (2.9–10.7)
	Lot	2.8 (1.1–7.2)
	Sample	0.50 (0.2–1.2)
**PCR method**	Feedlot	21.2 (14.7–29.5)
	Lot	20.1 (13.2–29.2)
	Sample	25.9 (17.5–36.6)

^a^ The proportions of samples that tested positive by culture and PCR methods were significantly different by McNemar’s Chi square test (*P* < 0.01).

^b^Kappa statistics: κ = 0.27; κ 95% CI = 0.21–0.32

^c^Kappa statistics: κ = 0.10; κ 95% CI = 0.04–0.17

### Characteristics of the *E*. *coli* O104 isolates

**Fig 1:**
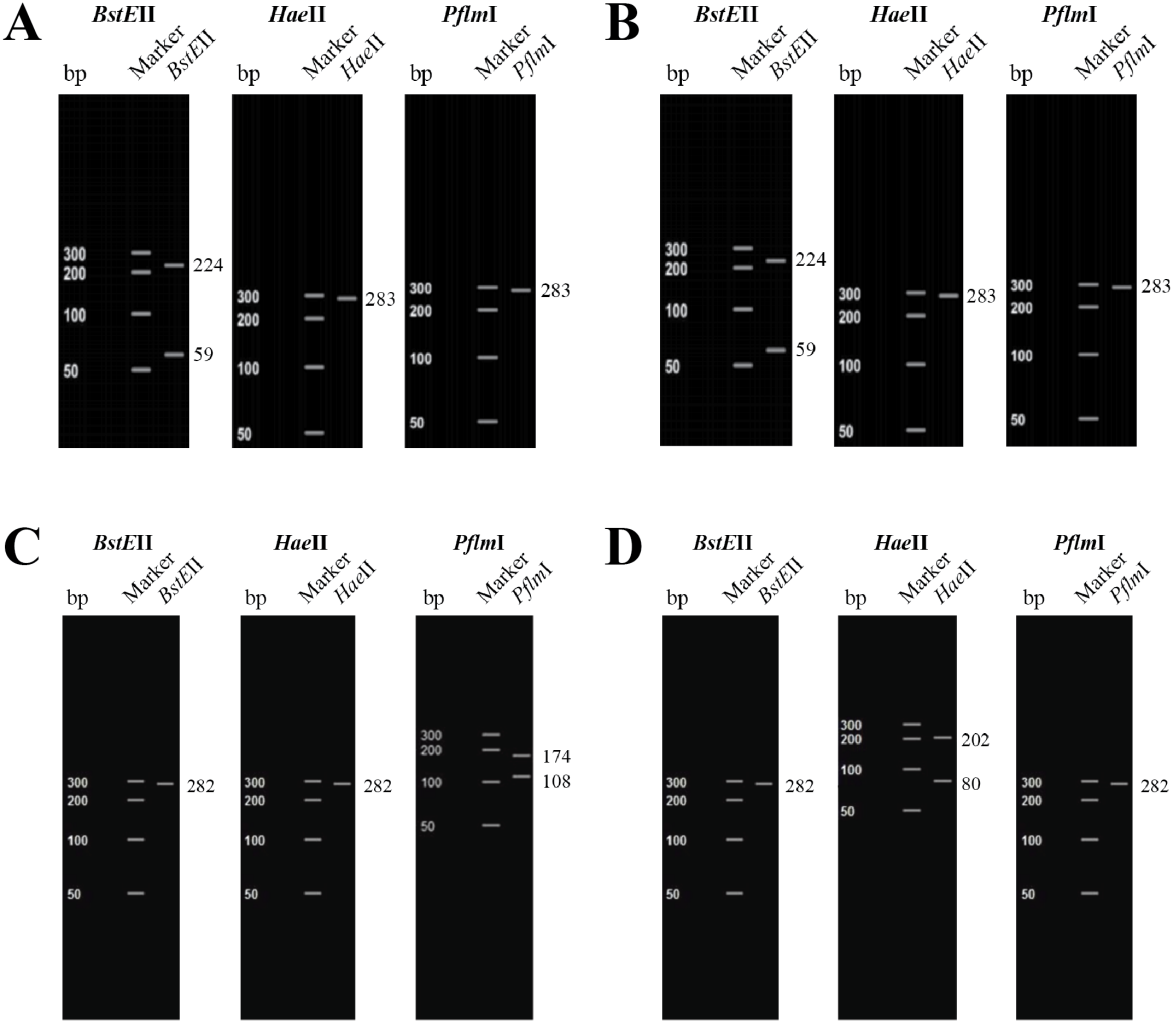
*In silico* restriction fragment length polymorphism (RFLP) subtyping of Shiga toxin genes of O104 isolates. (A) RFLP pattern of Shiga toxin of an O104 isolate; (B) RFLP pattern of *stx*1c of a reference sequence (Accession no. DQ449666.1); (C) RFLP pattern of *stx*1a of a reference sequence (Accession no. M16625.1); (D) RFLP pattern of *stx*1d of a reference sequence (Accession no. AY170851.1)

Of the 51 O104 isolates (positive for *wzx*_O104_ and negative for *wbdD*_O8/O9/O9a_), 16 isolates (31.4%) carried *stx*1 gene and all 16 also tested positive for *ehxA* and *terD* genes (Table 6). None of the O104 isolates carried *stx*2, *flic*_H4_, *eae*, *bfpA* and *aggA* genes (Table 6). Thirteen of the 16 *stx*1-positive O104 isolates were from one feedlot. Based on PCR assays targeting flagellar genes, 37 isolates tested positive for H7, four for H2, one each for H11 and H21, eight were unidentified, and none of the isolates tested positive for H4. The 16 *stx*1-positive O104 isolates possessed the H7 flagellar type. Amino acid sequences deduced from nucleotide sequences of *stx*1 amplicons indicated that the Shiga toxin genes of all O104 isolates (n = 16) were of subtype 1c. Digestion of *stx*1 amplicon (283 bp) with *BstE*II yielded two fragments of 224 and 59 bp whereas a single undigested fragment of 283 bp was produced with *Hae*II and *PflM*I enzymes. *In silico* RFLP analysis indicated that restriction patterns of Shiga toxin genes of O104 isolates were similar to that of *stx*1c of the reference strain (*E*. *coli* strain BCN26- Accession no. DQ449666.1; Fig 1) and matched the PCR-RFLP results (data not shown).

**Table 6:** Virulence gene profiles of strains of *Escherichia coli* O104 isolated from feedlot cattle feces.

				Genes				
Week of sample collection	Feedlot no.	No. of samples collected	Total no. of O104 isolates^a^	*stx*1	*stx*2	*eae*	*ehxA*	*terD*
**1**	2	38	2	1, 0	0	0	1, 0	1, 0
	2	19	1	1	0	0	1	1
	4	19	2	1, 0	0	0	1, 0	2
	4	19	5	0	0	0	0	0
	5	38	1	0	0	0	0	1
	6	19	3	0	0	0	0	0
	7	19	1	0	0	0	0	1
	11	19	3	0	0	0	0	1, 0, 0
	13	19	5	0	0	0	0	3, 0, 0
	14	16	2	0	0	0	0	1, 0
**2**	2	20	2	0	0	0	0	0
	2	20	1	0	0	0	0	0
	15	20	1	0	0	0	0	1
	17	20	1	0	0	0	0	0
	22	20	3	0	0	0	0	1, 0, 0
	22	20	3	0	0	0	0	0
	24	20	1	0	0	0	0	1
	26	20	9	9	0	0	9	9
	26	20	4	4	0	0	4	4
	27	20	1	0	0	0	0	0
	**Total**	**425**	**51**	**16**	**0**	**0**	**16**	**27**

^*a*^ Isolates positive for *wzx*_O104_ gene and negative for O8/O9/O9a

All isolates were negative for *bfpA*, *aggA* and *flic*_H4_ genes

### Pulsed-field gel electrophoresis (PFGE)

**Fig 2:**
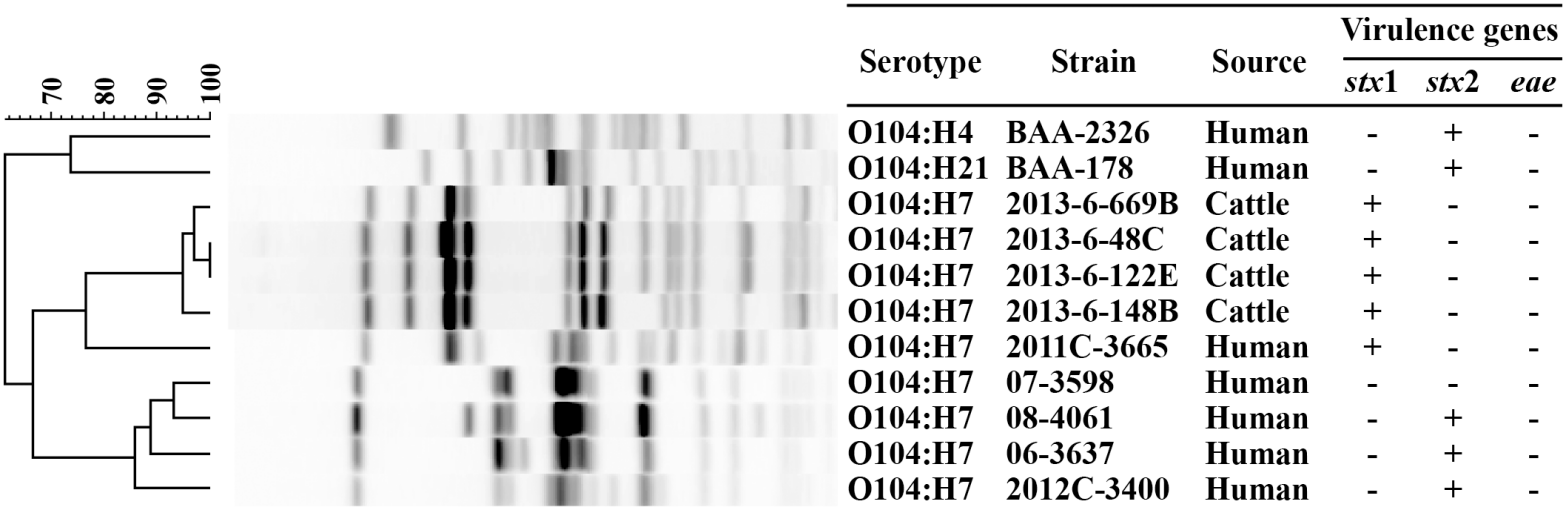
Pulsed-field gel electrophoresis-based clustering of *Escherichia coli* O104 strains from cattle feces and human clinical strains (O104:H4; O104:H21; and O104:H7).

The 16 *stx*1-positive *E*. *coli* O104 isolates obtained in the present study, German (O104:H4) and Montana outbreak (O104:H21) strains and the five human O104:H7 strains formed seven separate PFGE clusters (Fig 2). Bovine O104 strains were 62.4% similar to the German and Montana outbreak strains and 67.9% to 77.5% similar to human O104:H7 strains (Fig 2). The Dice similarity between the German outbreak and the Montana strains was 73%. The *stx*1-positive O104 strains (n = 13) obtained from the same feedlot were of the same PFGE subtype with 100% similarity, and the remaining three STEC O104 strains from different feedlots were of the same PFGE type (96–100% similarity).

## Discussion

The prevalence of *wzx*_O104_-positive *E*. *coli* was determined in feedlot cattle feces by culture and PCR methods of detection. Our primary goal was to detect the prevalence of serogroup O104. However, the target gene, *wzx*_O104_, used in the culture and PCR methods of detection is also present in *E*. *coli* O8, O9, and O9a serogroups [11]. In fact, the O antigen gene cluster of O104 serogroup has the same genes (O antigen polymerase gene, O antigen flippase gene [*wzx*_104_], three CMP-sialic acid synthesis genes, and three glycosyl transferase genes) in the same order as that of the gene cluster that codes for the K9 capsular antigen of O8, O9 and O9a serogroups [10, 11]. Therefore, a *wzx*_O104_-positive fecal sample could contain *E*. *coli* O104 and or O8/O9/O9a. In order to distinguish O104 from O8/O9/O9a, we assayed all fecal samples and pure cultures of *wzx*_104_-positive isolates by PCR with primers targeting *wbdD*_O8/O9/O9a_, a gene that is specific for O8/O9/O9a [11]. A fecal sample or an O104 isolate that was positive for *wzx*_104_ and negative for *wbdD*_O8/O9/O9a_ was considered as truly positive for serogroup O104.

The fecal samples collected in our study to estimate prevalence of *wzx*_O104_-positive *E*. *coli* were representative of a large population of cattle originating from 29 feedlots located in six Midwestern states. The prevalence of *wzx*_O104_-positive fecal sample, determined by PCR, reported in our study (46.1%) was higher than that reported (20.6%) by Paddock et al.[9], possibly because samples collected were from multiple feedlots (29 vs 8). Based on the culture method, 19.3% of fecal sample were positive for *E*. *coli* containing *wzx*_O104_ compared to 2.8% reported by Paddock et al [9]. In addition, the use of the O104-specific IMS beads likely increased the sensitivity of detection. None of the previous studies has utilized IMS beads because O104-serogroup specific beads had been commercially available only recently [19].

In the culture method, fecal samples that tested positive (pooled colonies) for *E*. *coli* possessing the *wzx*_O104_ gene indicated the sample was positive for O104 and/or O8.O9/O9a. However, isolates positive for *wzx*_O104_ and negative for *wbdD*_O8/O9/O9a_, which could be considered as truly *E*. *coli* O104, were obtained from 6.5% fecal samples. Ninety-two of the 143 *wzx*_O104_-positive isolates (64.3%) were also positive for *wbdD*_O8/O9/O9a_ by PCR, which means the isolates were not O104, but could be *E*. *coli* O8, O9, or O9a. Isolates positive for *wzx*_O104_ and *wbdD*_O8/O9/O9a_ have been reported in previous studies [9, 20]. Fecal prevalence of *E*. *coli* O8/O9/O9a has been previously reported in cattle [21, 22]. Manna et al. (2010) tested cattle feces collected at a slaughter plant in India for *E*. *coli* O8 and reported a prevalence of 2% [22]. Some of the chromogenic colonies picked from O104 beads-plated medium tested positive for other *E*. *coli* serogroups, such as O26, O45, O103, O145 and O157, which indicates some cross-reactivity of O104 beads with other serogroups. Unfortunately, the multiplex PCR targeting eight serogroup-specific genes (O104, O157 and 6 non-O157) that was used to test pooled colonies did not include the *wbdD*_O8/O9/O9a_ gene. Therefore, we could not ascertain the prevalence of serogroups of O8/O9/O9a in the pooled colonies. The detection of O157 and six non-O157 serogroups was low suggesting that non-specificity of the O104 beads does not appear to be an issue compared to IMS beads for other serogroups, particularly O103 [13]. We picked colonies with a range of color because chromogenic colonies of pure cultures of O104 serogroup on MP medium were indistinguishable from other serogroups of STEC

Our study showed that feedlot cattle harbor *E*. *coli* O104 serogroup (positive for *wzx*_O104_ and negative for *wbdD*_O8/O9/O9a_) in the gut and shed these organisms in their feces, however only a small proportion of the O104 isolates obtained carried the Shiga toxin gene and none exhibited the enteroaggregative genes of the pathotype of the German outbreak strain. Unlike other predominant STEC serogroups (O157 and non-O157 STEC) causing human illnesses, O104:H4 serotype has never been reported in animals. Previous studies that aimed at detecting the O104:H4 serotype carrying genes characteristic of the German outbreak strain in cattle feces reported that cattle do not harbor the combination of genes (*wzx*_O104_, *stx*1, *stx*2, *flic*_*H4*_, *aggA* or *agg*R) that are unique to this pathotype [7–9]. The present study confirms the absence of the unique pathotype in cattle feces in this population of feedlot cattle, based on both PCR and culture-based detection methods. *E*. *coli* O104 strains with H antigen, other than H4, have been isolated from animals [23–25]. Blanco et al. [23] have reported that O104:H7, positive for *stx*1 and negative for *stx*2 and *eae*, was one of the eight non-O157 serotypes more frequently detected among STEC strains in sheep in Spain, and interestingly, in the same study, none of the non-O157 STEC strains isolated from cattle included O104. Serotype O104:H21, positive for *stx*1 and *stx*2, but negative for *eae*, has been isolated from feces of healthy and diarrheic cattle in Spain [24]. None of the O104 isolates in our study tested positive for *stx*2 and the one isolate of H21 serotype obtained was negative for *stx*. To our knowledge, this is the first report of Shiga toxin carrying O104 serogroup in feces of cattle in the US.

Of the 51 O104 isolates (positive *wzx*_O104_ and negative for *wbdD*_O8/O9/O9a_), 16 (31.4%) carried a combination of *stx*1, *terD* and *ehxA* genes. Because the modified MP medium contained potassium tellurite, it is possible that there was a selection pressure exerted for *terD*-positive isolates. The *stx*1 of O104 isolates were of subtype *stx*1c based on nucleotide sequencing. Shiga toxin subtyping based on amino acid sequences were further confirmed by *in silico* RFLP, which matched results obtained from PCR-RFLP. Our study shows that *in silico* RFLP, a simple and rapid method, is a reliable alternative to PCR-RFLP for subtyping of *stx*. None of the O104 isolates obtained in the present study were positive for *eae*, indicating that serogroup O104 in our study population could be Shiga toxigenic, but not enterohemorrhagic *E*. *coli*. The absence of *eae* appears to be a feature of the serogroup O104 because previously reported serotypes such as O104:H4 (German outbreak strain; [2]), O104:H21 (Montana outbreak strain; [3]), and O104:H7 (CDC strains from sporadic diarrheal cases [5, 20] were all negative for *eae*. Based on PFGE typing, the O104:H7 strains of cattle origin were only 67.9% to 77.5% similar to human O104:H7 strains. Intimin-negative STEC isolates of serogroups O5, O76, O78, O113, O128, O146, O174, O178, and O181 carrying *stx*1c have been isolated from stools of asymptomatic carriers and individuals with diarrhea [26]. The O104 strains were also negative for *aggA* and *bfpA*, which are responsible for adherence to host cells in enteroaggregative *E*. *coli* [27] and enteropathogenic *E*. *coli* [28], respectively. All 16 O104 STEC strains isolated in our study carried H7 flagellar type and possessed the same profile of virulence genes tested (*ehxA* and *terD*). Miko et al. [5] have reported that STEC strains carrying same flagellar type generally harbor similar virulence genes. Thirteen of the 16 *stx*1-positive isolates were from the same feedlot and all 13 were of the same PFGE type, suggesting spread of a single clone within a feedlot.

*Escherichia coli* is a continuously evolving organism with the capacity to acquire virulence genes from other pathogenic organisms and become virulent [29]. Sialic acid which has been reported to be an important component of *E*. *coli* O104 antigen and other organisms such as *E*. *coli* O24, O56, *Campylobacter jejuni*, *Salmonella enterica*, and *Citrobacter freundii* [30, 31], is also an important component of animal tissues. This trait of bacterial antigens may contribute to evasion of immune system by mimicking the host tissue component [11]. Therefore, STEC O104:H7 serotype has the potential to be a human pathogen. Because the prevalence of O104 is low in cattle and only a small proportion of O104 is STEC, cattle are not likely to be a major reservoir for *E*. *coli* O104. *Escherichia coli* O104:H4 involved in the German outbreak in 2011 is a classic example of the emergence of a highly virulent pathogen by acquisition of prophage encoding Shiga toxin 2 through horizontal gene transfer [32]. Similarly, *E*. *coli* O104 with H types other than H4 has the potential to emerge as a virulent pathogen by acquiring Shiga toxins 1 and or 2 via phage-mediated transfer.

## Conclusions

Cattle harbor and shed *eae*-negative serogroup O104 in feces, however, none of the isolated strains in this study carried genes characteristic of the hybrid serotype reported in Germany (*stx*2, *aggA* and *flic*_H4_) and only a small proportion of O104 strains carried the *stx*1 gene. The predominant STEC serotype detected in cattle feces was O104:H7, which has been previously isolated from sporadic cases of diarrhea in humans. Based on our results, cattle are not a reservoir of O104:H4 serotype, however, they do harbor other O104 serotypes, such as O104:H2, O104:H7, O104:H11 and O104:21.
